# A comprehensive meta-analysis reveals the key variables and scope of seed defense priming

**DOI:** 10.3389/fpls.2023.1208449

**Published:** 2023-07-20

**Authors:** Lucia Talavera-Mateo, Alejandro Garcia, M. Estrella Santamaria

**Affiliations:** Centro de Biotecnología y Genómica de Plantas, Universidad Politécnica de Madrid – Instituto Nacional de Investigación y Tecnología Agraria y Alimentación, (UPM-INIA/CSIC), Madrid, Spain

**Keywords:** antagonist performance, plant fitness, plant performance, plant defense, priming agent, seed defense priming, biotic stress

## Abstract

**Background:**

When encountered with pathogens or herbivores, the activation of plant defense results in a penalty in plant fitness. Even though plant priming has the potential of enhancing resistance without fitness cost, hurdles such as mode of application of the priming agent or even detrimental effects in plant fitness have yet to be overcome. Here, we review and propose seed defense priming as an efficient and reliable approach for pathogen protection and pest management.

**Methods:**

Gathering all available experimental data to date, we evaluated the magnitude of the effect depending on plant host, antagonist class, arthropod feeding guild and type of priming agent, as well as the influence of parameter selection in measuring seed defense priming effect on plant and antagonist performance.

**Results:**

Seed defense priming enhances plant resistance while hindering antagonist performance and without a penalty in plant fitness. Specifically, it has a positive effect on crops and cereals, while negatively affecting fungi, bacteria and arthropods. Plant natural compounds and biological isolates have a stronger influence in plant and antagonist performance than synthetic chemicals and volatiles.

**Discussion:**

This is the first meta-analysis conducted evaluating the effect of seed defense priming against biotic stresses studying both plant and pest/pathogen performance. Here, we proved its efficacy in enhancing both, plant resistance and plant fitness, and its wide range of application. In addition, we offered insight into the selection of the most suitable priming agent and directed the focus of interest for novel research.

## Introduction

Plants are sessile organisms permanently restricted to their site of germination. They are constantly exposed to adverse biotic and abiotic environmental factors. To compensate the lack of mobility, plants have evolved several strategies to defend themselves against these threats. These defenses are costly to the plant because they divert energy and resources away from other plant processes as growth, development, or reproduction (Karasov et al., 2017). Specifically, in response to pathogens and pests, plants have evolved constitutive and inducible defenses (Freeman and Beattie, 2008). Constitutive or permanent defenses are constantly activated but they are not always needed, entailing high cost for the plants (Karban, 2011). In this sense, the plant defense theory suggests that inducible resistance has evolved to reduce the cost of constitutive defenses expression in the absence of antagonist (biotic stress), but still inducible defense has a penalty in plant growth, photosynthesis and/or reproduction (Huot et al., 2014; Garcia et al., 2021; He et al., 2022). To overtake these vulnerabilities, plants have developed the capacity to respond to previous stimuli by enhancing the activation of their inducible defenses upon later pathogen infection or herbivore attack (Frost et al., 2008; Martinez-Medina et al., 2016). This physiological process, known as defense priming, triggers a minor part of the plant defense response and prepares the plants to respond more quickly or aggressively to future biotic or abiotic stresses (Conrath, 2006). Besides, primed plants theoretically endure fewer costs relative to the direct activation of defenses, though still it might incur in some allocation and/or ecological costs (Frost et al., 2008), probably because it causes physiological alterations while shifting the plant to the alert (Conrath et al., 2015). Upon stimulus perception, changes occur in the plant at physiological, transcriptional, metabolic and epigenetic levels (Hilker and Schmülling, 2019). The condition of readiness achieved by the priming stimuli has been termed the *“Pre-challenge primed state”*. Upon subsequent challenge, the plant effectively mounts a faster and/or stronger defense response that defines the *“Post-challenge primed state”* and results in increased resistance and/or stress tolerance (Kanjariya and Parihar, 2017). To date, several priming agents have been applied to seedlings or adult plants to enhance plant response to bacteria or fungi infection, such as pathogen/microbe derived-stimuli (e.g., flg22, chitin, lipopolysaccharides, glucans), plant-growth-promoting rhizobacteria (e.g., *Pseudomonas putida*, *Burkholderia phytofirmans*), plant-growth-promoting fungi (e.g., *Fusarium* spp., *Trichoderma asperelloides*), natural or synthetic chemicals (e.g., beta aminobutyric acid [BABA], menadione sodium bisulfite [MSB], salicylic acid [SA], vitamins, hexanoic acid) and abiotic stimuli (e.g., heat, cold, wounding, UV) (Mauch-Mani et al., 2017). The effect of priming in plant response to herbivore attack has mostly focused on herbivore-induced plant volatiles (HIPV), which enhanced plant defense responses upon lepidopteran (Erb et al., 2015), mite (Agut et al., 2018) and aphid infestation (Sharma et al., 2017), herbivore feeding, oviposition, oral secretions or trichome sensing (Alborn et al., 1997; Alborn et al., 2007; Howe and Jander, 2008; Peiffer et al., 2009; Hilker and Meiners, 2010; Hilker and Fatouros, 2015; Ojeda-Martinez et al., 2021; Ojeda-Martinez et al., 2022). Silicon has also been tested as a priming agent in adult plants via soil fertilizer or foliar spray to improve plant response to different arthropods, with no consistent evidence of having a greater effect in any particular feeding guild or class (Keeping and Kvedaras, 2008; Reynolds et al., 2016).

Most of these works on defense priming have been performed applying the priming stimuli to seedlings or adult plants. However, it has been recently suggested that the success of priming against herbivores can be improved if the priming agent is applied to the seeds (Westman et al., 2019). Seed defense priming (SDP) may confer long lasting protection from an early age and promises to be both, labor and resource-efficient, poses minimal environmental risk, and is, thus, economically and ecologically promising. In addition, it has been described that the effects produced by SDP are not transitory and can persist throughout the plant life cycle, being transmitted to the next generations in a process termed *“immunological memory”* (Lal et al., 2018). Even though the system appears commercially attractive and profitable at the economic and ecological level, the published evidence is scarce. It has been suggested that this could be the consequence of private companies driving most of the research (Pedrini et al., 2017). There are some works where SDP has been tested against pathogens and herbivores (Pushpalatha et al., 2011; Nagaraju et al., 2012; Worrall et al., 2012; Zeng et al., 2012; Hamada and Jonsson, 2013; Jogaiah et al., 2013; Smart et al., 2013; Castillo Lopez et al., 2014; Strapasson et al., 2014; Babu et al., 2015; Berglund et al., 2016; Song et al., 2017; Hamada et al., 2018; Mouden et al., 2020; Maurya et al., 2019). These studies used different priming agents (bacteria, fungi, plant hormones, vitamins, volatiles….) applied to seeds to improve crops, cereals, herbaceous or tree resistance to different pathogens or pests.

**Figure 1:**
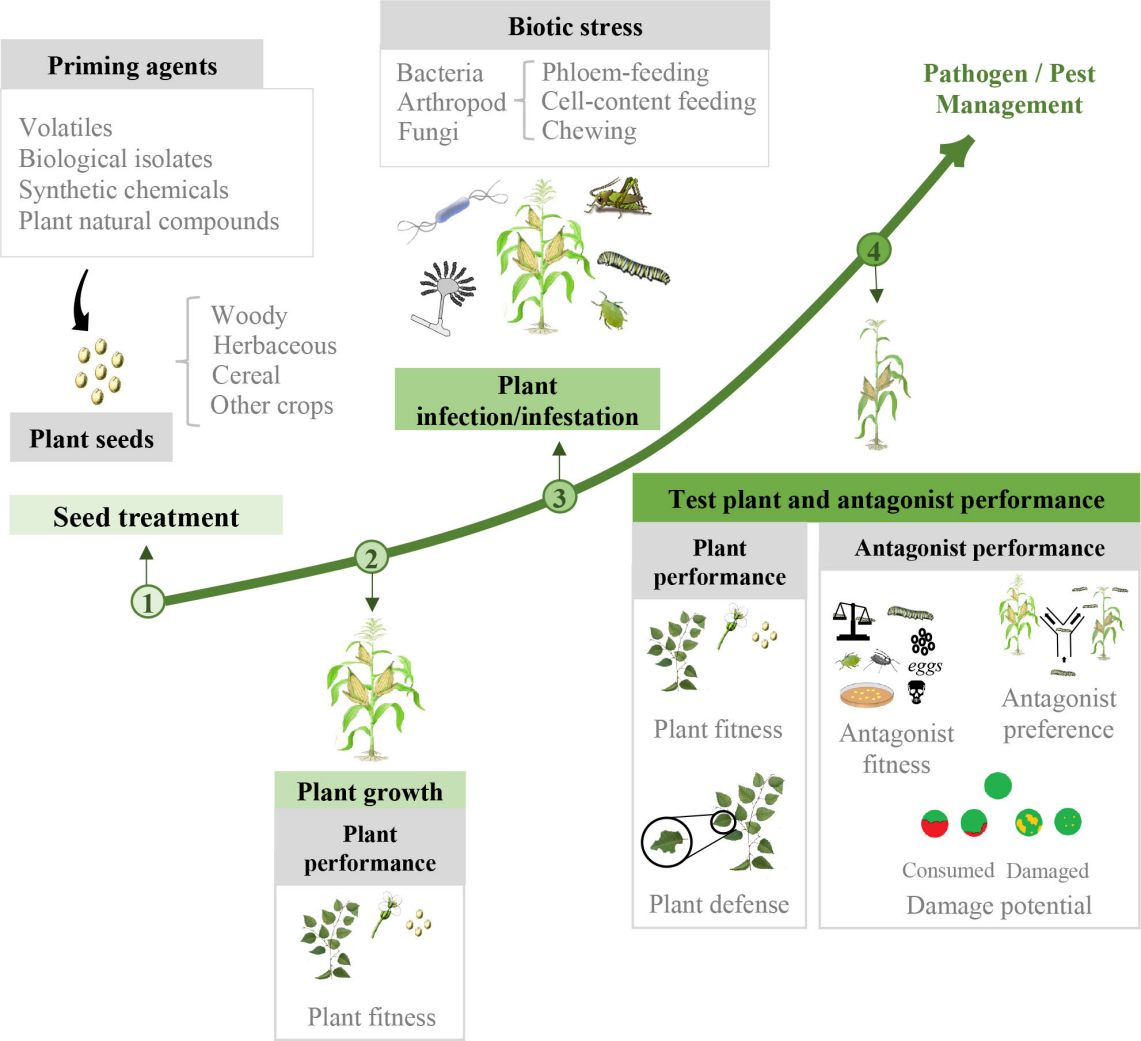
Diagram depicting SDP process with variables evaluated at each time-point. (1) Seed treatment: priming agents are applied to seeds. (2) Plant growth: plant fitness parameters are measured to study SDP effect on plant growth before applying stress. (3) Plant infection/infestation: primed and control plants are challenged against biotic stress. (4) Plant and antagonist performance parameters are evaluated for each organism.

To have an overview of the potential of SDP in pathogen and pest management, in this meta-analysis we analyzed plant and antagonist response in a comprehensive way. We collect all the available studies in which priming agents are applied to seeds to later evaluate plant and antagonist performance upon biotic stress. The effects of the priming agent are studied, including both resistance and fitness perspectives, and having into account different types of priming agents, host plants and antagonist classes (**Figure 1**). The importance of the different variables in the final success is discussed.

## Materials and methods

We designed a systematic review protocol following the Preferred Reporting Items for Systematic Reviews and Meta-Analyses (PRISMA; Moher et al., 2009). This allowed a rigorous compilation of information on the following questions explored in this meta-analysis: (i) Does SDP have an effect on plant and antagonist performance upon biotic stress? (ii) Are the effects observed in plant performance after the stress inversely correlated with antagonist behavior? (iii) Are the parameters used to measure plant and antagonist performance appropriate to determine the final effect? (iv) Does the enhancement of plant resistance match with an improvement on plant fitness? (v) Is the wingspan of the effect dependent on the plant host or the antagonist class? (vi) Is the success of SDP related with the feeding guild of the arthropod? (vi) Is the breadth of the effect dependent on the type of priming agent used? (vii) Is the magnitude of this effect related to the parameter used to estimate plant or antagonist performance?

### Compilation of the database

A literature search was conducted to collect all relevant published data, with no restriction of publication date, related to the effect of seed priming on plant resistance. In order to differentiate whole papers and specific experiments within those papers, herein after “publication” refers to papers while “study” refers to experiments. The publication screening process (i.e. PRISMA flow diagram) is provided in **Supplementary Data Figure S1**. Selection was performed via online databases such as Google Scholar, Pubmed and ScienceDirect by a combination of keyword searches including “seed priming”, “plant priming”, “plant defense”, “biotic stress” and “plant resistance”. Following this, an abstract and title screening was performed, excluding publications which do not contemplate outcomes related to the effect of priming on plant defense, studies where the priming agent was not applied to seeds, studies at other developmental stages, and studies where the effect of seed priming was analyzed against abiotic stress. Additional publications were also retrieved by examining the references of the only two SDP reviews published to date (Westman et al., 2019; Yang et al., 2022).

### Database building

Experimental design and result communication vary greatly across different publications. This makes it imperative to create a yardstick to be applied in order to include only studies that can be comparable and that contribute with a similar weight to the meta-analysis. The eligibility criteria in terms of inclusion/exclusion enforced were as follows: 1) studies include a treatment of seeds with an elicitor or agent that is quantifiable and reproducible, 2) the agent is applied individually, 3) a combination of priming agents is allowed when they belong to the same category out of the 4 included in our classification (plant natural compounds, synthetic chemicals, biological isolates or plant volatiles), 4) no post- or any other complementary treatment is applied once the seeds germinate, 5) plants are challenged against a biotic stress i.e. antagonist (pathogens – fungus, bacteria – or pests – arthropods), 6) stress is applied as a singular event at a specific time-point with no combination of treatments or posterior re-application, 7) both treatments (priming and stress) have an appropriate control carried along the whole experiment, 8) performance parameters are measured at specified time points, 9) all data necessary to calculate effect sizes is provided (n, mean, standard error or standard deviation of both control and treatment, 10) when more than one treatment is studied, data of all treatments is extractable and 11) image resolution allows a confident reading of the results obtained.

An extensive amount of studies was extracted and filtered as valid data for the meta-analysis. Nevertheless, due to the lack of raw data comparing different treatments with different purposes, a main classification was implemented distinguishing treatment effect on plant and treatment effect on antagonist. Furthermore, several subdivisions were established to fully explore the effect of priming on both plant and antagonist. All categories studied in this meta-analysis are reflected in **Figure 1**. Within plant performance, two main separate aspects were considered: plant fitness and plant defense. Plant fitness and surrogated parameters are measured before and after stress application (e.g. plant height, fruit yield). Plant defense includes quantification of non-damaged plant tissue (e.g. remaining leaf area after feeding). Effect on plant performance was also evaluated from a species point of view, classifying plant species in 4 groups: woody, herbaceous, cereals and crops (although cereals could be considered crops, these 2 categories were separated as there was a high number of studies focused only on cereals while several others used different types of agronomical important plants that could not be defined as a major group other than crops). Within antagonist performance, 3 main separate aspects were considered: antagonist fitness, antagonist preference and antagonist damage potential. Antagonist fitness includes population and propagation parameters (e.g. larval weight, mycelial growth). Antagonist preference includes acceptance and feeding/oviposition preference experiments, which was only applicable for pests (e.g. antixenosis). Antagonist damage potential includes quantification of the damage caused to the plant (e.g. disease severity, feeding damage). Effect on antagonist performance was also evaluated from an organism point of view, classifying biotic stress in 3 groups: fungus, bacteria and arthropods. Additionally, due to the large amount of studies including arthropods, these were categorized according to their feeding guild into: cell-content suckers, phloem-feeders and chewers. Finally, in order to more deeply examine the effect of the different priming agents applied to seeds, their impact on both plant and antagonist performance was studied depending on their chemical nature, biological involvement and origin, and therefore classified as: plant natural compounds (jasmonic acid [JA], SA, methyl jasmonate [MeJA], BABA and plant vitamins), synthetic chemicals (CaCl_2_, INA, nucleoside analogues, benzothiadiazole), biological isolates (chitosan and bacterial and fungal isolates) and plant volatiles. All the different categories explored in this meta-analysis and their associated publications can be found in **Supplementary Data Table S1**.

### Data extraction

Sample sizes, mean values and variances or standard errors of each study were extracted from the corresponding tables and figures. The online free tool PlotDigitizer (https://plotdigitizer.com/) was used to extract data from graphs and plots in numerical format. If image resolution was too low the study was excluded. Each agent concentration tested was considered as an individual study, whether effect was positive, negative or neutral. Studies missing standard error or variance data were excluded. When sample size was given as a range of values, the lower one was used for effect size calculation. In studies including resistant and susceptible plant cultivars only the susceptible one was selected for the meta-analysis as representation of the plant species.

### Effect sizes analysis

The meta-analysis was conducted using R software v4.1.1 (R Core Team, 2021) and RStudio v2022.07.1 + 554 software (RStudio Team, 2022). Effect sizes were calculated as Hedge’s *g* to apply a small sample bias correction to the standardized mean difference by Cohen’s *d* (Hedges, 1981). All analyses were performed using the “metacont” function from the “meta” package (Balduzzi et al., 2019) for pooling effect sizes with a random-effects model to account for between-study heterogeneity (*τ^2^*) and with a restricted maximum likelihood (Veroniki et al., 2016). In addition, a Knapp-Hartung adjustment was applied to calculate the confidence interval around the pooled effect reducing the risk of a false positive result, since it accounts for the uncertainty of the between-study heterogeneity (Knapp and Hartung, 2003).

The magnitude of the treatment was considered to be statistically significant when the 95% confidence interval (CI) of the effect size did not overlap with 0. When analyzing plant parameters, effect sizes statistically higher than 0 mean a positive effect of the priming agent on plant performance. When analyzing antagonist parameters, effect sizes statistically lower than 0 mean a negative effect of the priming agent on antagonist performance.

A quantification and assessment of the between-study heterogeneity was performed in order to accomplish a better comprehension of the validity, data quality and robustness of our effect sizes estimations. The Higgins and Thompson’s *I^2^* (Higgins and Thompson, 2002) and *τ^2^* (IntHout et al., 2016) statistics were used as indicators of the heterogeneity variance.

With the objective of reducing heterogeneity and providing more certainty of the robustness of the pooled effect sizes, outlier and influential case detection was conducted using the “find.outliers” function from the “dmetar” package (Harrer et al., 2019). After outlier identification and removal, all effect sizes were then computed. *I^2^* and *τ^2^* were once again calculated to account for the between-study heterogeneity of our final effect sizes.

Finally, to further strengthen the validity of the conclusions extracted from this meta-analysis, a publication bias risk assessment was conducted. With this, we aimed to address the fact that study publications are not totally objective and there is a varying probability of getting published depending on their results (Page et al., 2021). Moreover, bias may also exist due to questionable research practices, being *p*-hacking one of the most outstanding ones (Simonsohn et al., 2014). For each effect size, both reporting bias and *p*-hacking were tackled including a fail-safe number and a *p*-curve analysis, respectively. Fail-safe numbers (n_fs_) were calculated using the weighted method of Rosenberg (2005). *P*-curve analyses were performed with the “pcurve” function from the “dmetar” package.

### Statistical analyses

Mean effect sizes were compared to study the similarities and differences throughout the categories explored in this meta-analysis. 95% confidence intervals (CIs) of statistically significant pooled effect sizes were considered as a proxy of significance (Nakagawa and Cuthill, 2007). When overlapping of CIs occurred between two categories, a T-test was performed.

## Results

### Meta-analysis data

A total of 6206 publications were initially identified, 6181 came from searching the databases Google Scholar, Pubmed and ScienceDirect, and 25 came from the references cited in papers (**Supplementary Data Figure S1**). After duplicates and no defense-related titled papers were removed, 1247 articles remained for abstract screening, of which 140 were selected for retrieval. Finally, 62 were identified as relevant publications. After a thorough analysis, 3 of them were excluded for applying the priming agent to seedlings or adult plants, 14 of them were excluded for studying plant defense against abiotic stress, 10 of them were excluded due to lack of necessary data and 11 of them were excluded for not contributing with experimental data (i.e. reviews). Therefore, a total number of 26 publications fitted our selection yardstick. These articles were comprehensively examined and individual experiments were extracted following the 7 criteria previously mentioned, resulting in a total of 350 studies included in this meta-analysis. Out of these 350 performance measurements, 146 belonged to plant parameters and 204 to antagonist parameters.

A further dissection of the selected studies allowed us to establish the following variables as partially explanatory of SDP effect on plants: (1) plant performance, (2) plant host, and (3) priming agent. Likewise, the following variables were established as partially explanatory of SDP effect on antagonists: (1) antagonist performance, (2) class, (3) feeding guild within arthropods, and (4) priming agent. These variables led to the formation of different subgroups (**Supplementary Data Dataset S1**).

Heterogeneity was analyzed across all different categories as a proxy for robustness and data quality. An overview is given in **Supplementary Data Table S1**. The presence of a general moderate between-study heterogeneity can be explained due to the specificities of each publication included in this meta-analysis, which employ different plant species, different biotic stresses and different priming agents. Grouping of variables allowed the homogenization of the data which, along with the lack of publication bias observed by the significant fail-safe numbers and the *p*-curve analyses (**Supplementary Data Figures S2, S3**), provide enough reliability and robustness to the validity of the results.

### SDP has an effect on both plant and antagonist performance

**Figure 2:**
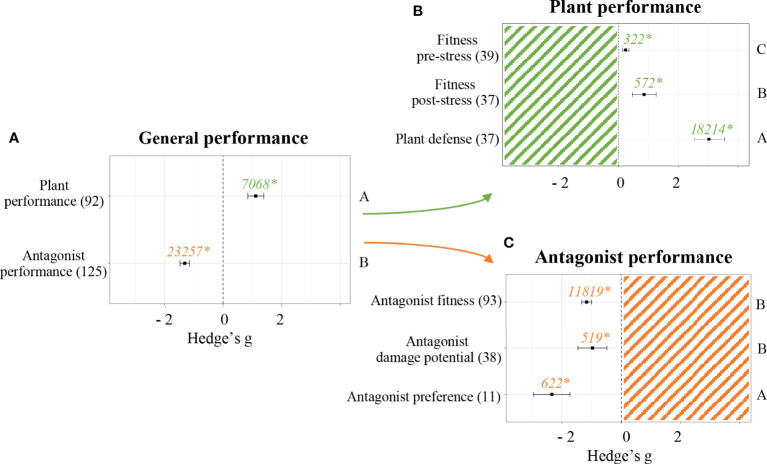
Effect of seed defense priming on plant and antagonist performance. Subgroups included general organism performance **(A)**, plant performance parameters **(B)** and antagonist performance parameters **(C)**. Sample sizes are provided in brackets. Symbols indicate pooled values of Hedge’s g with their 95% CI. Negative and positive values indicate a negative and positive effect of SDP on the corresponding organism, respectively. Different letters indicate significant differences between subgroups (*p*-value < 0.05). Rosenberg’s fail-safe numbers are reported in italics along with an asterisk indicating significance.

Performance parameters were evaluated to assess whether the application of a priming stimulant on seeds resulted in phenotypical differences in either the plant or the antagonist it was challenged against. The experiments included in this meta-analysis indicated SDP had different effects depending on the categories of the parameters studied. The two main categories evaluated were parameters related to plant and antagonist performance. In both cases, SDP showed significant differences (*p* < 0.05, **Figure 2A**). Positive effects were detected when measuring plant performance parameters (Hedge’s g = 1.1136 ± 0.27, n = 92). Negative effects were detected when measuring antagonist performance parameters (Hedge’s g = -1.3104 ± 0.16, n = 125).

Effect sizes of experiments accounting for plant defense were separated from effect sizes of experiments accounting for plant fitness pre-stress application and post-stress application in order to obtain a better understanding of how SDP affects plant performance (**Figure 2B**). In all cases, a significant positive effect was found (*p* < 0.05). Plant defense was more heavily influenced by SDP than plant fitness, with Hedge’s g = 3.0495 ± 0.52 (n = 37). Then, fitness parameters measured after the plant was challenged against a biotic stress showed a more positive influence from SDP than fitness parameters measured before stress application, being Hedge’s g = 0. 8585 ± 0.4 (n =37) and Hedge’s g = 0.2432 ± 0.11 (n = 39), respectively. Since SDP had a general positive effect on fitness, following analyses were performed with the variable “fitness” accounting for both pre- and post-stress application parameters, which allowed to reach more robust conclusions.

Likewise, effect sizes of experiments accounting for antagonist fitness were separated from those accounting for antagonist preference and from those accounting for antagonist damage potential in order to obtain a better understanding of how SDP affects antagonist performance (**Figure 2C**). The effects were significantly negative in all cases (*p* < 0.05). The most negative effects were detected for antagonist preference (Hedge’s g = -2.3354 ± 0.62, n = 11), equally followed by antagonist fitness (Hedge’s g = -1.1650 ± 0.16, n = 93) and antagonist damage potential (Hedge’s g = -0.9708 ± 0.5, n = 38).

### SDP effect magnitude depends on host plant and antagonist class

**Figure 3:**
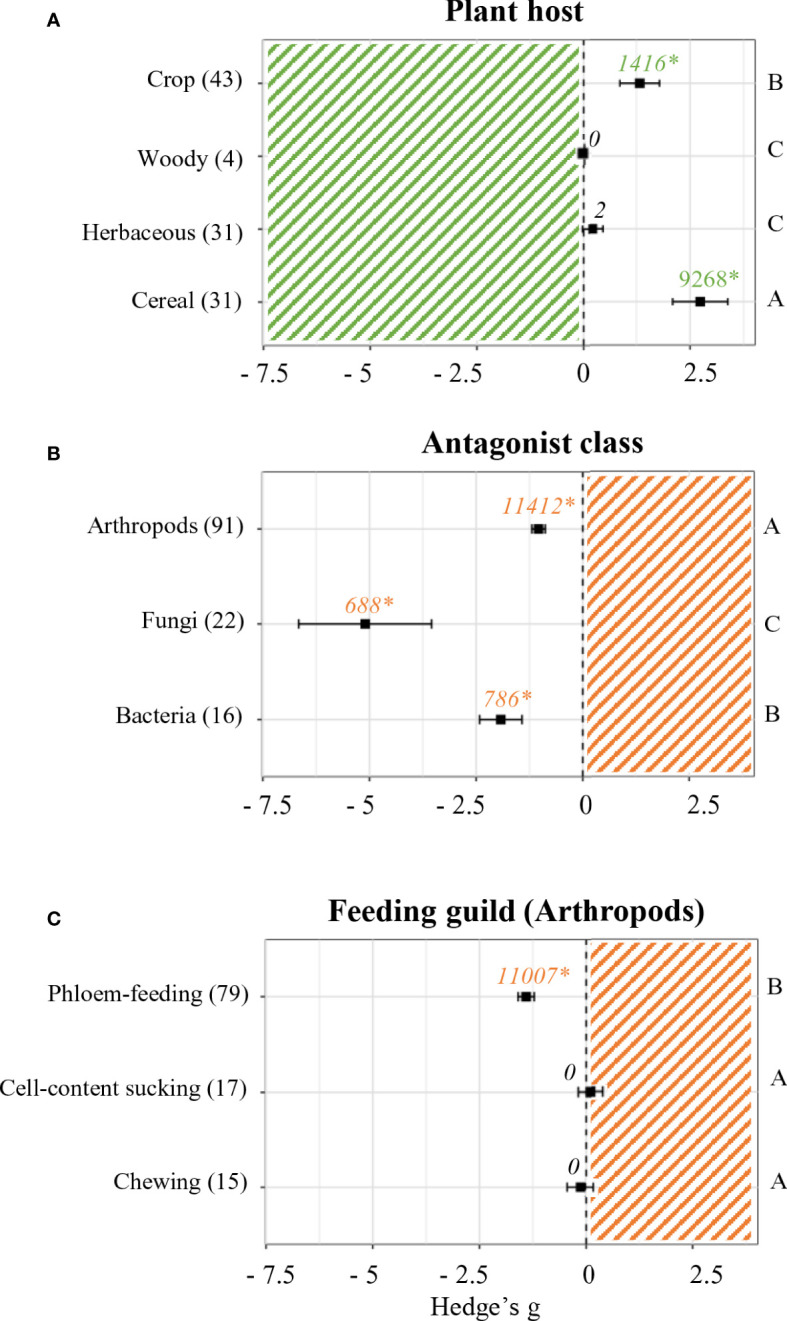
Effect sizes on plant and antagonist performance parameters classified by subgroups. Subgroups included plant host **(A)**, antagonist class **(B)** and feeding guild **(C)**. Sample sizes are provided in brackets. Symbols indicate pooled values of Hedge’s g with their 95% CI. Negative and positive values indicate a negative and positive effect of SDP on the corresponding organism, respectively. Different letters indicate significant differences between subgroups (*p*-value < 0.05). Rosenberg’s fail-safe numbers are reported in italics along with an asterisk indicating significance.

Plant performance effect sizes were also analyzed depending on the plant host (**Figure 3A**). While no effects were accounted for in regards of woody (n = 4) and herbaceous plants (n = 31), positive significant effects were found in experiments performed on cereals and crops (*p* < 0.05). The most positive effects were detected for cereals (Hedge’s g = 2.7364 ± 0.66, n = 31), followed by crops (Hedge’s g = 1.3269 ± 0.48, n = 43).

Similarly, experiments performed with different classes of biotic stress were separated and compared (**Figure 3B**). SDP had a negative effect on all organisms (*p* < 0.05). The most negative effects were found in studies that included fungi as antagonist (Hedge’s g = -5.0999 ± 1.58, n = 22), followed by those that used bacteria (Hedge’s g = -1.9208 ± 0.5, n = 16) and lastly those that challenged primed plants against arthropods (Hedge’s g = -1.0.372 ± 0.16, n = 91).

Due to the availability of a large number of studies where arthropods were used as biotic stress, a deeper analysis of effect sizes was performed within this group by differentiating feeding guilds (**Figure 3C**). Out of the 3 feeding guilds identified, only 1 was significantly affected by SDP (*p* < 0.05). Neither arthropods that feed by chewing (n = 15) nor those that feed by sucking cell content (n = 17) showed differences in their performance. On the other hand, experiments performed with phloem-feeding arthropods did show a significantly negative mean effect size (Hedge’s g = -1.4075 ± 0.1, n = 79).

### SDP success depends on the nature of the priming agent

**Figure 4:**
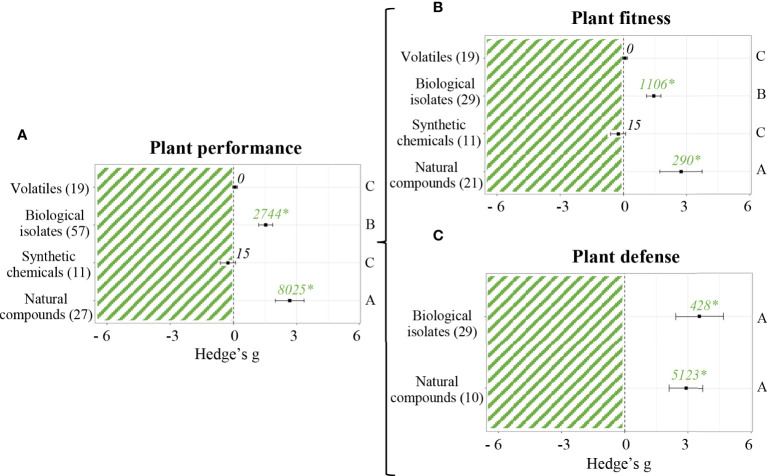
Effect sizes of priming agents on plant performance classified by subgroups. Subgroups included general performance **(A)**, plant fitness (pre- and post-stress application) **(B)** and plant defense **(C)**. Sample sizes are provided in brackets. Symbols indicate pooled values of Hedge’s g with their 95% CI. Positive values indicate a positive effect of SDP on the corresponding organism. Different letters indicate significant differences between subgroups (*p*-value < 0.05). Rosenberg’s fail-safe numbers are reported in italics along with an asterisk indicating significance.

A total of 33 different molecules were used as priming agents among the 350 studies included in this meta-analysis. Agents were classified in 4 groups in order to analyse their differential effect on plant and antagonist. Thus, within each group, studies were further separated into 2 subcategories depending on the parameters measured (i.e. plant performance or antagonist performance parameters). **Figure 4A** shows differences among types of stimulants when measuring plant performance parameters. Neither synthetic chemicals nor volatiles did affect plant performance. All other agents did have a positive effect (*p* < 0.05). Biological isolates affected plant performance (Hedge’s g = 1.5282 ± 0.34, n = 57), but plant natural compounds (Hedge’s g = 2.6669 ± 0.68, n = 27) did have a stronger positive effect. A more accurate analysis was performed to study the influence of each type of priming agent on plant fitness (**Figure 4B**) and plant defense (**Figure 4C**), separately. Likewise, the prior was mainly affected by natural compounds (Hedge’s g = 2.7352 ± 1.01, n = 21) and biological isolates (Hedge’s g = 1.4401 ± 0.34, n = 29), while synthetic chemicals and volatiles did not have any effect. The later only included studies performed with either biological isolates or plant natural compounds, and both of them rendered positive effects on plant defense (Hedge’s g = 3.5561 ± 1.13, n = 29 and Hedge’s g = 2.9130 ± 0.81, n = 10, respectively). There was no information regarding plant defense parameters of primed plants treated with synthetic chemicals or volatiles.

**Figure 5:**
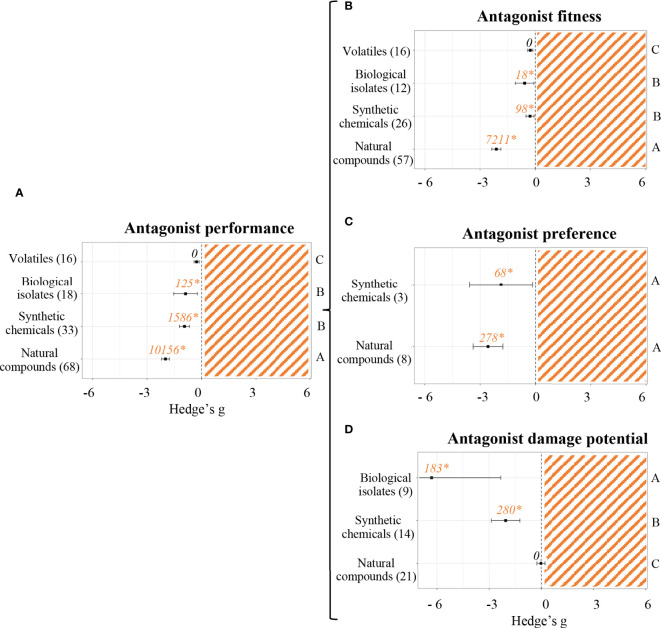
Effect sizes of priming agents on antagonist performance classified by subgroups. Subgroups included general performance **(A)**, antagonist fitness **(B)**, antagonist preference **(C)** and antagonist damage potential **(D)**. Sample sizes are provided in brackets. Symbols indicate pooled values of Hedge’s g with their 95% CI. Negative values indicate a negative effect of SDP on the corresponding organism. Different letters indicate significant differences between subgroups (*p*-value < 0.05). Rosenberg’s fail-safe numbers are reported in italics along with an asterisk indicating significance.

**Figure 5A** shows differences between types of priming agents when measuring antagonist performance parameters. Once again, volatiles did not prove to have any effect. All other priming agents did render a negative effect (*p* < 0.05). The most vigorous effects on antagonist performance were caused by plant natural compounds (Hedge’s g = -1.9838 ± 0.21, n = 68), followed by synthetic chemicals (Hedge’s g = -0.9386 ± 0.27, n = 33) and biological isolates (Hedge’s g = -0.8804 ± 0.65, n = 18). The differential effect of each group of priming agents on antagonist fitness (**Figure 5B**), antagonist preference (**Figure 5C**) and antagonist damage potential (**Figure 5D**) was also evaluated. Antagonist fitness was most negatively affected by plant natural compounds (Hedge’s g = -2.1167 ± 0.25, n = 57). Although on a much weaker magnitude, synthetic chemicals (Hedge’s g = -0.2850 ± 0.22, n = 26) and biological isolates (Hedge’s g = -0.5779 ± 0.5, n = 12) did also have a negative effect on antagonist fitness, whereas volatiles (n = 16) did not show any effect whatsoever. Antagonist preference parameters were only evaluated in experiments using either natural compounds or synthetic chemicals as priming agents. Both cases rendered significant results, being Hedge’s g = -2.5737 ± 0.81 (n = 8) and Hedge’s g = -1.8672 ± 1.7 (n = 3), respectively. Lastly, antagonist damage potential parameters were measured in studies where biological isolates, synthetic chemicals or plant natural compounds were used as priming agents. Natural compounds (n = 21) did not seem to have any effect, whereas biological isolates and synthetic chemicals reduced the damage caused by antagonist to primed plants (Hedge’s g = -6.3030 ± 3.97, n = 9 and Hedge’s g = -2.0503 ± 0.82, n = 14, respectively).

## Discussion

### SDP causes an overall impact in plant and pest performance

SDP is expected to increase plant resistance while minimizing the penalty in fitness. This meta-analysis supports that SDP confers plants an enhanced defense system. It has been mentioned that defense priming can incur cost due to resource allocation towards defense activation rather than plant growth and reproduction (Vos et al., 2013) and according to Martinez-Medina et al. (2016), there is a small penalty in fitness right after applying the priming agent. Here, we investigated separately the impact of SDP in plant defense and in plant fitness, concluding that SDP is not only capable of hijacking the defense-growth trade-off and improving plant resistance without a cost in plant fitness, but also, in some cases, primed plants even display better fitness. In accordance with Martinez-Medina et al. (2016), SDP resulted in an enhancement of the plant defensive response which, ultimately, led to a benefit in plant fitness. Moreover, we separated plant fitness parameters measured before challenging plants against a biotic stress from those measured after stress application. In contrast with what would be expected, SDP proved to have a positive effect on plant fitness in the early stages, when plants had not been under stress conditions and fitness benefit was not supposed to be reached. This would violate the cost-benefit balance where maintenance of the “*primed state*” requires an initial penalty in fitness that is later compensated with a more robust defense response against the antagonist, resulting in better plant performance and fitness benefit. However, the studies included in this meta-analysis register no common agreement as to which and when parameters are measured. This heterogeneity in timing points and protocols, reflected quantitatively as well by the between-study heterogeneity results obtained, could be masking the real effect of SDP on those early moments after applying the priming agent. Furthermore, different plant fitness and surrogate parameters might not reflect the same magnitude of effect, as proven by Garcia et al. (2021). Likewise, the same meta-analysis showed how the plant ontogenetic stage and the magnitude of infestation might affect treatment success. In SDP, these factors would likely play a role in how long primed plants take to make up for the initial fitness penalty. Thus, as it was already addressed by Westman et al. (2019) regarding defense priming, it is imperative to establish more rigid guidelines as to “which” and “when” parameters should be evaluated, especially when measuring the initial fitness costs. Nevertheless, Martinez-Medina et al. (2016)’s model was developed with defense priming applied to seedlings and adult plants as reference, and might not be adequate for SDP. One way or another, it is compelling the fact that some studies did show a fitness benefit in treated plants even before challenging them against biotic stresses. In a deeper analysis of these occurrences when applying SDP, we recognized vitamins and SA as the key players (Pushpalatha et al., 2007; Pushpalatha et al., 2011; Mahesh et al., 2017). In contrast, Cipollini (2002) showed that the exogenous application of SA to foliage inhibited plant growth, which supports SDP treatments’ mode of application directly to seeds.

Antagonist performance parameters have only been measured in individual experiments, but no generalization has yet been studied. In this meta-analysis, we explored how SDP influences antagonist behavior by separating impact on their fitness, the actual damage caused by the antagonist and their host preference. SDP does not seem to have a specific antagonist target but rather causes a negative overall effect in fitness and damage potential with a similar magnitude. Interestingly, preference assays, which were only studied challenging primed plants against arthropod herbivores, did render a more acute response to SDP, suggesting that molecular differences between naïve/untreated and primed plants might be perceived by arthropods “at first sight”. Initially, the most likely candidate to play the main role in such a task would be JA or its conjugated forms (Howe, 2004). However, no experiments have been performed yet where arthropod preferences were tested against plants coming from seeds primed with JA, indicating that a totally different molecular pathway originated in thiamine could be undergoing (Hamada and Jonsson, 2013; Hamada et al., 2018).

### SDP has a wide spectrum of application in the field

There is ample evidence that a large number of agents can induce a vigilant physiological state (Tiwari and Singh, 2021). Nevertheless, not all priming agents render similar results when applied to different hosts or challenged against different antagonists. Cereals and crops in general come across as the most suited plant hosts when applying SDP, which would be incredibly profitable for agriculture and the development of novel environmentally-friendly pest management strategies (Desmedt et al., 2021). Nevertheless, woody plants cannot be classified as poor host for SDP due to the scarce amount of research. It is curious to notice that herbaceous plants do not appear to be significantly impacted by SDP when measuring plant performance parameters, however, all antagonist classes see their performance reduced. Mishra et al. (2018) studied priming herbaceous plants against fungi and found little to no differences in plant fitness but a significant drop in seedling mortality rate after pathogen infection. All other experiments with herbaceous plants were performed applying volatiles as priming agents, which rendered no effects of treatment whatsoever. These molecules showed no effect either on plant or antagonist performance. The lack of significant impact when using volatiles could be concealing the real effect of SDP on herbaceous plants. Thus, research on herbaceous plants, using any priming agents other than volatiles, is required in order to make a better assessment of SDP success in this case.

One of the most notable discoveries of this meta-analysis is the efficacy of SDP against all classes of antagonist included in this work. Although studies of SDP as a defense mechanism against viruses were not found, recent research has showed that a pre-exposition of plants to pathogens (Jeon et al., 2017), chemical treatments (Ando et al., 2021) or volatiles (Su et al., 2020) conferred an enhanced resistance against a secondary infection in the case of viruses. It is well known that, when it comes to plant defense, even though, in general hormones play a major role (Bari and Jones, 2009), plants resort to different strategies depending on whether they are faced against herbivores (Santamaria et al., 2013), fungi (Ferreira et al., 2007) or bacteria (Melotto et al., 2006). Here we prove that SDP is a valid tool against all of them, although not all organisms are equally affected. Arthropods seem to be less sensitive to SDP than fungi or bacteria, even though it is the antagonist class most studied so far. It would be appealing to invest in fine-tuning proper concentration and mode of application for each arthropod species in order to develop more suitable and effective SDP treatments against herbivores. Nonetheless, the negative effect of SDP on arthropod performance is very conclusive and its apparently lower scope in comparison with pathogens could simply be due to the low number of pathogen-related studies, which is also reflected by the higher between-study heterogeneity in pathogen analyses. In addition, arthropod’s feeding guild appears to have an influence in SDP effect. Depending on the feeding mode of action, plant hormones differentially regulate induced defenses. JA regulates induced defenses against chewing insects (Schmiesing et al., 2016), whereas SA regulates defenses against phloem-feeding insects (Thaler et al., 2012). Induced defenses against mesophyll sucking mites are regulated by both JA and SA (Zhurov et al., 2014; Santamaría et al., 2017; Santamaría et al., 2019). Owing to the few studies published so far, no further examination could be performed regarding the differential effect of plant hormone molecules against each specific feeding guild, but it would be highly interesting for future analyses to compare the individual effect of JA and SA as priming agents on arthropods depending on their feeding modes. It seems curious that out of the 3 feeding guilds studied in this meta-analysis only phloem feeders appeared to be affected by SDP when, as a whole category, arthropods were negatively impacted. This could be easily explained by the fact that cell-content suckers and chewers were only tested against a few priming agents, whereas studies with phloem-feeding arthropods included the whole range. Furthermore, chewing arthropods were tested against volatiles (Maurya et al., 2019) or using woody plant species (Berglund et al., 2016), thus, the lack of effect is probably due to the plant host and the priming agents applied. Once again, the disparate number of experiments between groups hinders a definite conclusion and stresses the need for further research, mainly on arthropods belonging to the cell-content sucking and chewing feeding guilds.

### SDP specificity and scope

This meta-analysis compared influences of different types of priming agents on plant and antagonist performance. Out of the 4 groups generated, biological isolates was the only one studied across all antagonist classes and rendered positive results. However, even though plant natural compounds were not tested against bacteria, this group performed remarkably well against arthropods and fungi, making it worth of further study. In this context, hormonal crosstalk must be addressed. It is only reasonable to question whether the use of priming agents with opposite defense response pathways, would be detrimental for the primed plant when challenged against the non-targeted antagonist. According to Vos et al. (2015), a combination of treatments does not necessarily cause additional negative effects. This prompts that the activation of a specific defense pathway through SDP against a certain type of biotic stress, does not automatically reduce the plant defensive capabilities against a different type of antagonist. In fact, we identified several studies where they applied the same priming agent indistinctly of the biotic stress. For example, jasmonates are successfully used against biotrophs (Worrall et al., 2012), hemibiotrophs (Król et al., 2015) and necrotrophs (Kępczyńska and Król, 2012). Westman et al. (2019), in their meta-analysis of defense priming applied to seedlings or adult plants, highlighted the efficiency of using molecules such as BABA, oligogalacturonides, jasmonates (which would belong to our “plant natural compounds” category) as priming agents, although they doubted the validity of MeJA due to the potential detrimental effect on plant phenotype. Contrastingly, in a more recent publication by Yang et al. (2022), this one focused on SDP, several studies were included where MeJA was used as a priming agent exhibiting positive results. This renders the question of whether MeJA might not be an ideal candidate for defense priming on seedlings and adult plants, but, on the contrary, could be a completely sound priming agent to be used on seeds.

### SDP *vs* defense priming applied to other plant tissues

Previous to this work, there was only 1 meta-analysis that studied the effect of priming plants with the objective of enhancing resistance to biotic stresses. This meta-analysis, conducted by Westman et al. (2019), included studies where priming was applied to either foliage or root tissue. Interestingly, they proved that fungal isolates and vitamins (which belong to the "plant natural compounds" category) were the most effective priming agents, and that arthropods seemed to be less sensitive to primed plants than fungi or bacteria. Both statements highlight similarities between seed, shoot and root priming. Nonetheless, whereas defense priming applied to adult plants (either foliage or root tissue) was most effective against bacteria, here we found that SDP affects fungi more heavily. Defense priming has persistently demonstrated its efficacy as pest and pathogen management tool. However, as aforementioned, mode and tissue of application and plant developmental stage seem to be pivotal when determining the efficacy of a specific priming agent. In addition to prior advocacy for SDP (Lal et al., 2018; Westman et al., 2019; Yang et al., 2022), this meta-analysis attests and strengthens its success in enhancing not only plant defense, but also plant performance. Thus, we propose SDP as the most cost-effective, eco-friendly and agronomically viable approach.

## Conclusions

This meta-analysis offers some insight into one of the most innovative and promising disease control and pest management strategies for crop protection. The last few years have been pivotal for SDP research, with an exponentially growing number of publications. The integration of all public knowledge to date has allowed us to confirm that (i) SDP has a positive effect on plant performance, at the same time that causes a negative effect on antagonist performance; (ii) SDP does not seem to undergo the initial penalty in fitness due to resource allocation that happens when priming is applied to seedlings or adult plants; (iii) SDP seems to affect more positively to cereals and crops than woody and herbaceous plants; (iv) SDP is likely to grant plants a more powerful resistance against fungi than against bacteria and herbivores; (v) SDP appears to have a differential effect on arthropods depending on their feeding guild, being a useful protection tool against phloem-feeders; (vi) Different priming agents confer different magnitude of resistance, being plant natural compounds and biological isolates the best candidates as SDP agents.

Since SDP is a relatively new approach, further research is needed in order to establish detailed protocols and knowledge about seed priming effect on commercially relevant crop species, from agent concentration and mode of application to effect reliability and long-term stability. This work highlights the potential of SDP as a crop protection tool to be implemented in agricultural systems and stresses the key points to be addressed in upcoming studies.

## Data availability statement

The original contributions presented in the study are included in the article/**Supplementary Material**. Further inquiries can be directed to the corresponding author.

## Author contributions

MS conceived the idea. MS and LT-M designed the study, conducted the literature search and analyzed the data. LT-M and AG performed the manuscript figures. MS and LT-M wrote the original draft of the manuscript. AG made substantial contributions to enhance a final version of the manuscript. All authors read and approved the final manuscript.
