# Supplementary material for: A comprehensive meta-analysis reveals the key variables and scope of seed defense priming
Source: Front Plant Sci. 2023 Jul 20;14:1208449. doi: 10.3389/fpls.2023.1208449 (PMC10398571; doi:10.3389/fpls.2023.1208449)
Supplement: Supplementary file 2 [file DataSheet_1.pdf]

Supplementary Material

A comprehensive meta-analysis reveals the key variables and scope of Seed Defence Priming

Lucia Talavera-Mateo[1], Alejandro Garcia[1], and M. Estrella Santamaria[1]*

[1] Centro de Biotecnología y Genómica de Plantas, Universidad Politécnica de Madrid – Instituto Nacional de Investigación y Tecnología Agraria y Alimentación, (UPM-INIA/CSIC), Madrid, Spain

*Correspondence:

Dra. M. Estrella Santamaria

me.santamaria@upm.es

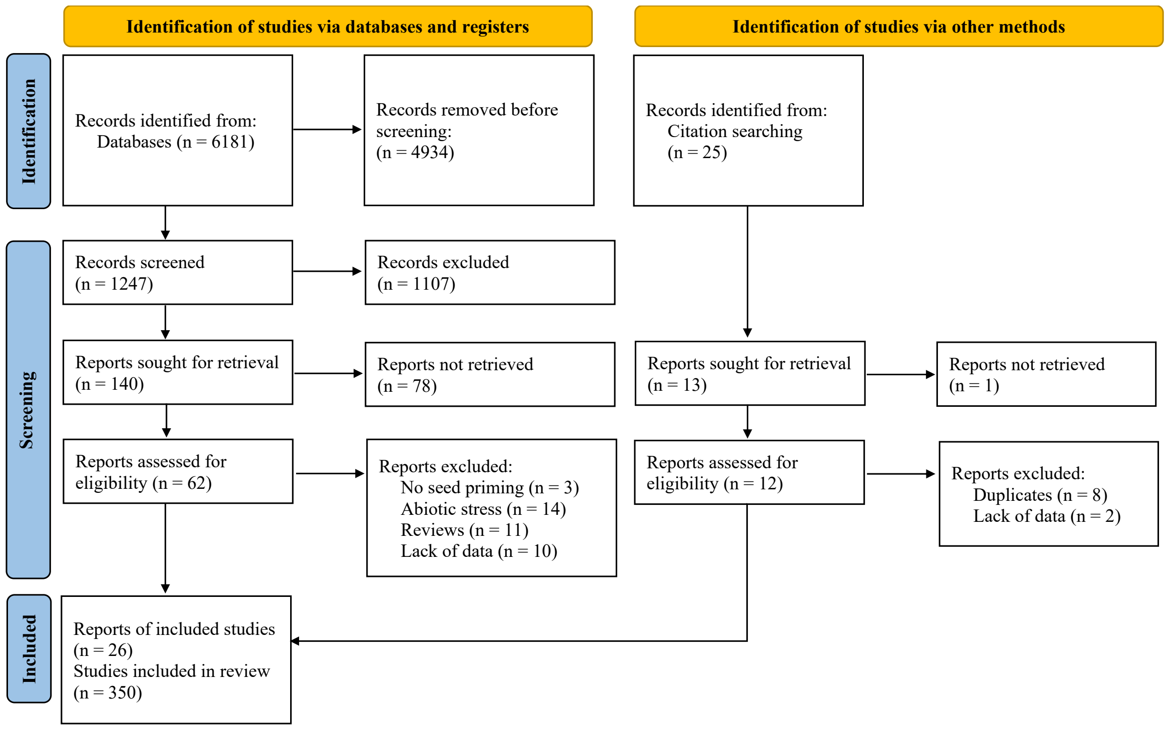

Supplementary Figure 1. PRISMA flow diagram detailing the screening process followed for the inclusion/exclusion of publications in the meta-analysis. Whole papers are referred to as “records” or “reports”, whereas the individual experiments performed within papers as referred to as “studies”.

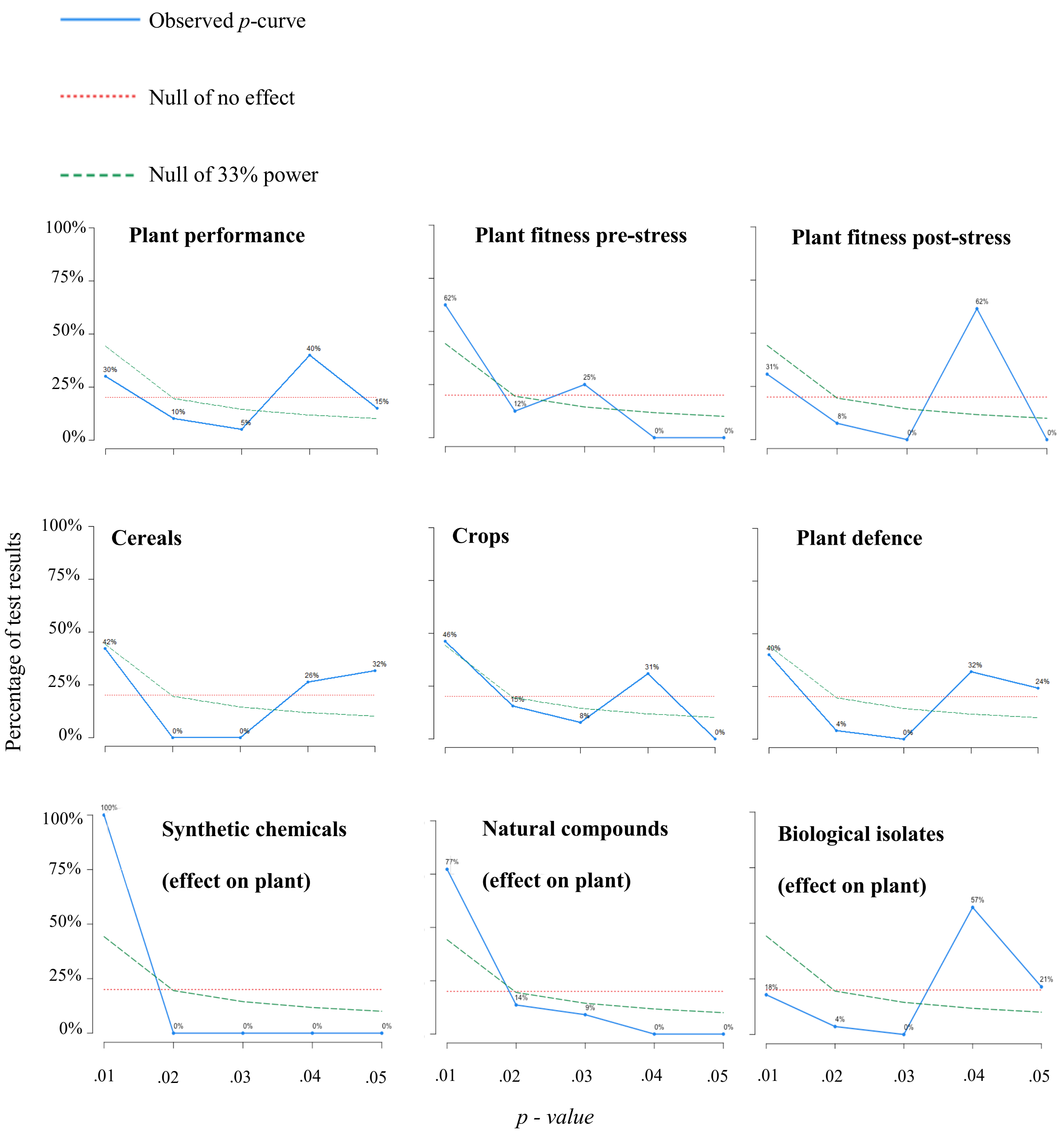

Supplementary Figure 2. P-curves of each plant category analysed in this meta-analysis.

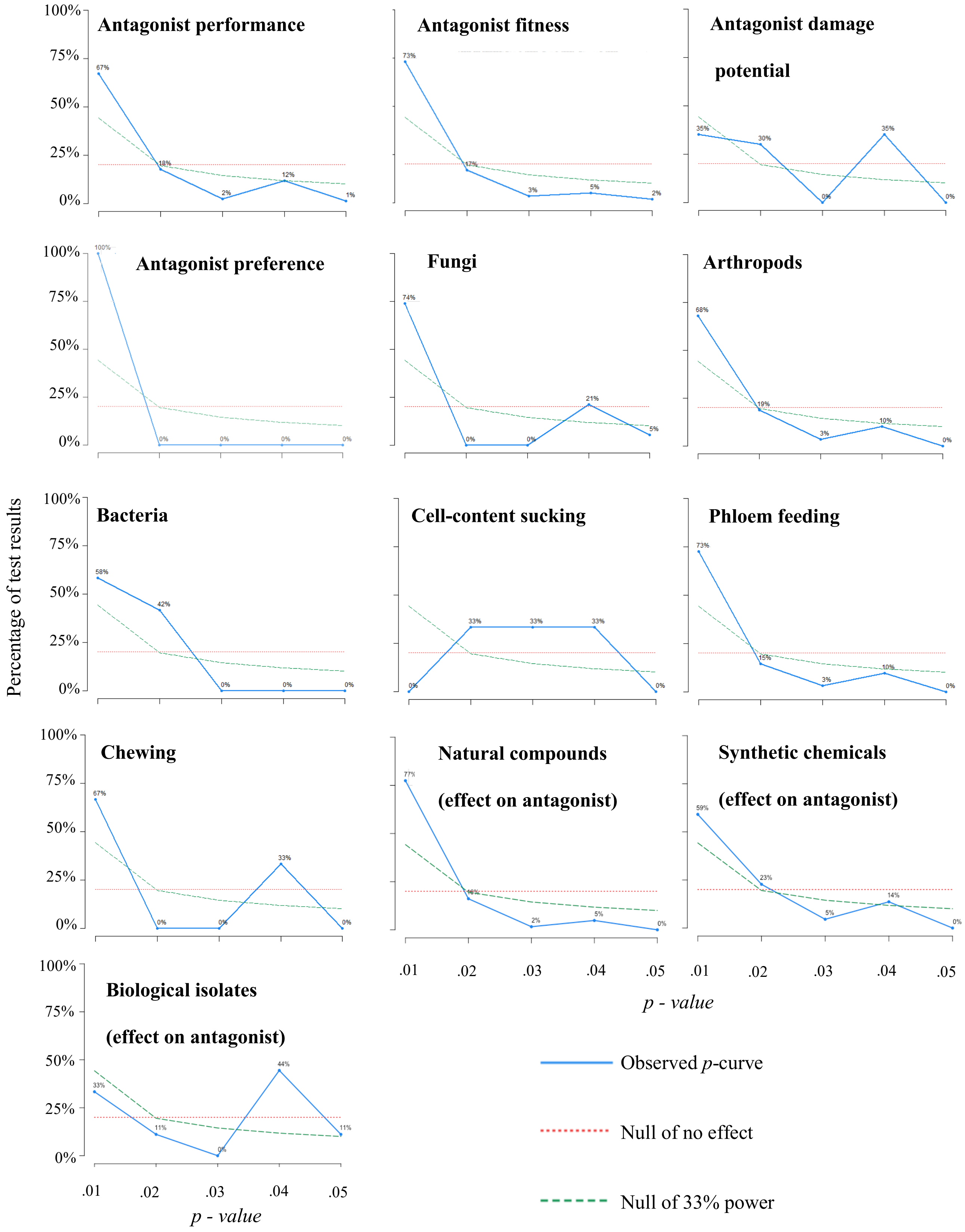

Supplementary Figure 3. P-curves of each antagonist category analysed in this meta-analysis.

Supplementary Table 1. Between-study heterogeneity parameters of each category analysed in this meta-analysis.

Category		Number of studies	Number of publications	tau^2	I^2 (%)
Plant performance		146	13	0.3666	55.3
Antagonist performance		204	19	0.2675	52.5
Plant fitness pre-stress		60	6	0.0196	19.38
Plant fitness post-stress		42	3	0.3915	61.98
Plant defence		44	7	0.612	56.4
Woody		4	1	0	0
Cereal		31	3	1.0098	79.5
Crop		43	7	0.5186	63.2
Herbaceous		31	2	< 0.001	17.8
Antagonist fitness		138	13	0.184	47.8
Antagonist preference		11	3	0.463	65.9
Antagonist damage potential		55	7	0.8381	76.2
Fungi		22	7	6.8184	68.2
Arthropod		91	9	0.1836	51.7
Bacteria		16	3	0.3854	54.3
Cell-content sucking		17	2	0.0385	23.3
Phloem-feeding		79	6	0.2344	50.2
Chewing		15	3	0.1195	45.4
Plant	Natural compounds	51	6	1.3431	88.89
	Synthetic chemicals	13	2	0.2765	63.66
	Biological isolates	62	6	0.1117	40.24
	Volatiles	20	1	0	0
Antagonist	Natural compounds	113	9	0.3233	46.11
	Synthetic chemicals	53	6	0.2565	72.41
	Biological isolates	22	5	0.3927	60.27
	Volatiles	16	1	0	0
